# Investigating the key influencing factors of pre-jump height in juvenile trampoline gymnasts using grey relational analysis

**DOI:** 10.3389/fpsyg.2025.1596942

**Published:** 2025-07-29

**Authors:** Hui Wang, Su Zhang, Hanya Dai, Mingxin Gong, Ningxiang Zou, Feng Jia, Lejun Wang

**Affiliations:** ^1^Sport and Health Research Center, Shanghai YangZhi Rehabilitation Hospital (Shanghai Sunshine Rehabilitation Center), Physical Education Department, Tongji University, Shanghai, China; ^2^Memorial Hall of the First National Congress of the Communist Party of China, Shanghai, China

**Keywords:** juvenile trampoline gymnasts, pre-jump height, influencing factors, grey relational analysis, factor analysis

## Abstract

**Objective:**

Achieving greater pre-jump elevation is essential for trampoline gymnasts to attain high-level competition performance. This study aimed to explore the key influencing factors of pre-jump height in juvenile trampoline gymnasts, thereby providing a rationale for training programs and talent selection criteria.

**Methods:**

A mixed-methods approach was adopted. First, a comprehensive literature review, expert interviews, and theoretical analysis were conducted to establish preliminary indicators potentially influencing pre-jump height. Second, experimental tests were carried out on 16 juvenile trampoline gymnasts to collect data on these indicators. Third, factor analysis was applied to refine the initial indicator system and develop a formal evaluation framework. Finally, grey relational analysis was used to quantify the relationships between each indicator and pre-jump height.

**Results:**

The final indicator system encompassed 5 dimensions and 16 representative variables. The grey relational analysis revealed that 10 indicators—standing long jump, height, leg length, shoulder width, arm hang angle, ratio of counter-jump height to pre-jump height, 30-s hanging leg raise, BMI, hip joint angle at landing, and state anxiety level—showed strong correlations (grey relational coefficient > 0.9) with pre-jump height. Furthermore, three additional indicators—trampoline-induced acrophobia, daily acrophobia, and ankle joint cushioning—demonstrated moderate correlations (coefficient 0.8–0.9). In contrast, time perception (10 s), supine leg raise (45°), and self-rotation perception showed weaker correlations (coefficient < 0.8).

**Conclusion:**

This study established an indicator system consisting of 16 items of pre-jump height influencing factors and identified the importance ranking of each indicator using grey relational analysis in juvenile trampoline gymnasts. These findings may serve as a scientific basis for developing targeted training programs and objective talent selection criteria while advancing gymnastics research by highlighting the interaction of physical, technical, and psychological factors in specialized jumping performance.

## 1 Introduction

Trampoline is a competitive sport known as the “ballet in the air” that combines artistry, aesthetics, watchability, and entertainment (Aydin et al., 2023). Since being introduced as an official event in the Olympic Games in 2004, the popularity and attention surrounding trampoline gymnastics have steadily increased. In trampoline exercise, before performing a 10-element routine technique, athletes first achieve optimal pre-jump height through several pre-jumps (Aalizadeh, 2014; Dyas et al., 2023). A higher pre-jump height not only increases the time of flight score but also provides more time in the air for the completion of the technique (de Oliveira et al., 2021). Therefore, achieving the maximum pre-jump height is very important for trampoline athletes to complete the latter 10-skill routine and achieve excellent competition results (Fang, 2016; Hu, 2018). Our previous study on the grey correlation between the sub-item scores and the total score of excellent trampoline athletes also found that the contribution of the height score to the total score is much higher than that of other sub-item scores (Wang et al., 2018). In the daily training of juvenile trampoline gymnasts, training and improving pre-jump height is a central concern for coaches (Sands et al., 2019).

During the execution of pre-jumps, athletes primarily generate kinetic energy through repeated active extensions of the hip, knee, and ankle joints in the lower limbs, combined with coordinated arm swinging. They convert and utilize the elastic potential energy from the trampoline bed through proper technical movements, thereby achieving the ideal pre-jump height (Sands et al., 2019). Therefore, the pre-jump height of trampoline athletes is influenced by various factors such as anthropometry, lower limb strength, specialized techniques, and psychological quality (Everhart et al., 2020). Investigating the relationship between these influencing factors and pre-jump height can provide a theoretical basis and guidance for the selection and scientific training of juvenile trampoline gymnasts. However, the specific impact of different influencing factors on the pre-jump height of juvenile trampoline gymnasts still remains unclear.

Grey relational analysis (GRA) is a method used to reveal the degree of mutual influence among factors or the extent of contribution of factors to the main behavior (Nayakappa, 2019). It can intuitively and simply address issues related to the evaluation and ranking of multiple factors' effects, as well as quantitative analysis problems. GRA is one of the most commonly used methods for quantifying the contribution rates of factors (Pradhan, 2017; Chen et al., 2022). This study aimed to explore the key influencing factors of pre-jump height in juvenile trampoline gymnasts based on grey relational analysis, thereby providing a scientific basis for designing targeted training programs and objective criteria for talent identification, as well as contributing to the broader field of gymnastics by highlighting the interplay between physical, technical, and psychological attributes in specialized jumping performance.

## 2 Materials and methods

### 2.1 Participants

A total of 16 gymnasts (age 13.0 ± 2.0 years; height 152.7 ± 10.3 cm; weight 40.7 ± 8.6 kg) from the Shanghai Gymnastics Center participated in this study. All participants met the following inclusion criteria: (1) at least 4 years of experience in specialized trampoline training; (2) no history of neuromuscular disorders, no acute viral infections (e.g., influenza, COVID-19) within 2 weeks prior to testing, and no musculoskeletal injuries in the past 6 months; (3) avoidance of strenuous exercise for 48 h prior to testing to prevent residual fatigue; and (4) adherence to an 8-h fasting period and 24-h caffeine abstinence before physiological measurements. The participants were fully informed of the experimental procedures and provided written assent (parental consent for minors). The study was approved by the Ethics Committee of Tongji University (No. 2020TJDX006).

### 2.2 Study design

**Figure 1:**
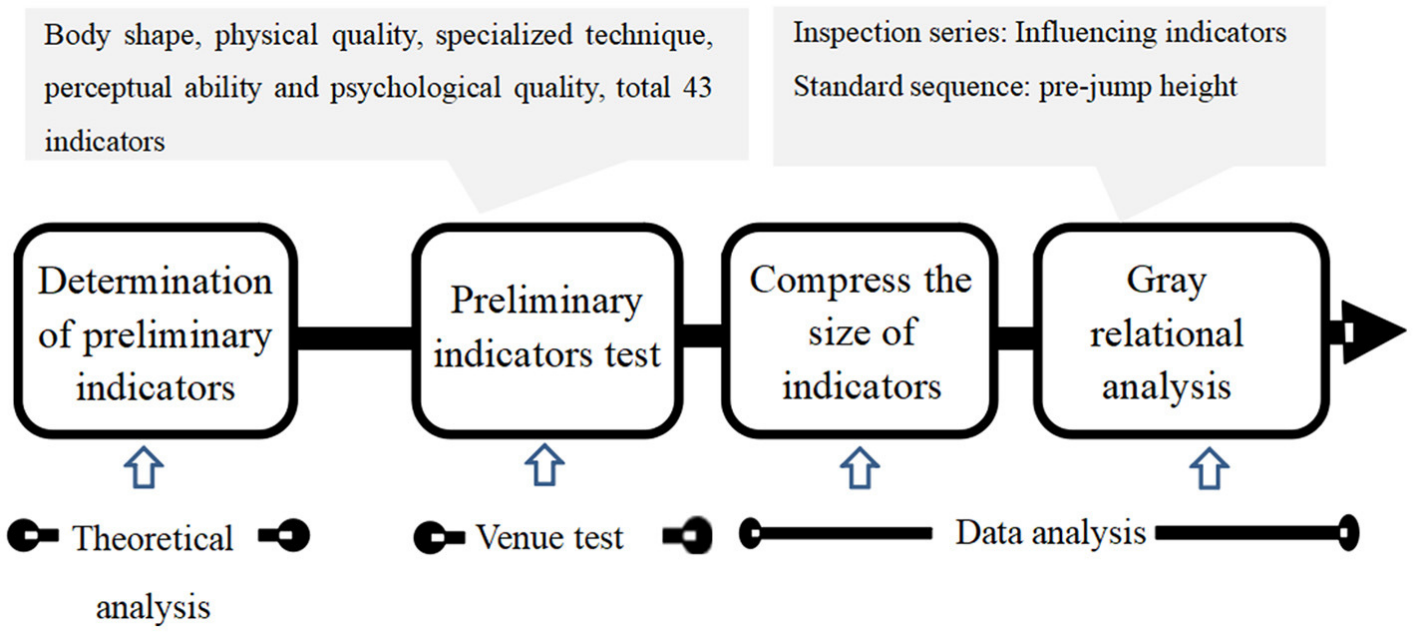
Research protocol.

The protocol of the current study included four procedures: (1) Constructing a preliminary set of indicators influencing the pre-jump height of juvenile trampoline gymnasts; (2) testing the preliminary set of indicators on juvenile trampoline gymnasts to obtain the test results of the set; (3) based on the empirical data from the tests, using factor analysis to streamline the secondary indicators under each primary set of predictive indicators in the preliminary set of indicators, thereby forming a set of predictive indicators for the influencing factors of pre-jump height in juvenile trampoline gymnasts; and (4) selecting the pre-jump height as the standard sequence and each predictive indicator as the inspection series and using grey relational analysis to calculate the grey relational coefficient between the pre-jump height and each predictive indicator, thereby quantifying the impact of each predictive indicator on the pre-jump height. The research protocol is shown in Figure 1.

### 2.3 Construction of the preliminary set of indicators

Through theoretical analysis of trampoline sports and a review of previous research on factors influencing pre-jump height, potential influencing factors of pre-jump height were obtained, and a 5-level questionnaire to assess the importance of each potential influencing factor was developed (Thung et al., 2021). Trampoline coaches and trampoline technique researchers from the Sports Science Research Institute were surveyed as experts to score each item on the questionnaire. The items with an average score higher than 3.5 points and for which consensus on importance was reached among the experts were selected, and the preliminary set of indicators influencing pre-jump height was determined.

### 2.4 Experimental testing and data processing of the preliminary set of indicators

The test was conducted at the trampoline training hall of the Shanghai Gymnastics Sports Center. The test indicators included four dimensions of anthropometry, physical quality, specialized techniques, and psychological quality, totaling 43 indicators.

The formal test procedure consisted of three distinct phases, each separated by a 72-h interval. Before the formal test, the participants were familiarized with all experimental procedures and test movements through standardized demonstrations. During the first phase, the participants underwent anthropometry measurements, including height, weight, BMI, leg length, upper limb length, foot length, thigh circumference, calf circumference, chest circumference, waist circumference, shoulder width, hip width, and arm span. Following the anthropometry test, a standardized 5-min rest period was implemented before commencing the physical quality assessments.

Prior to the physical quality test, the participants engaged in a structured 20-min warm-up protocol incorporating stretching exercises, jogging, and submaximal jumping activities. The physical quality testing followed a scientifically designed sequence that progressed from static to dynamic assessments to optimize performance and safety. The test began with static flexibility measurements, including sit-and-reach and arm hang angle assessments. Subsequent evaluations focused on explosive items, including counter jump, squat, rebound after a high landing, and standing long jump tests. The physical quality testing concluded with strength endurance assessments, which included the 30-s hanging leg raise, 30-s burpees, 30-s back extension test, and 30-s sit-up and reach test. To ensure methodological rigor, the sequence of specific test variations within each assessment category was randomized across participants. Scientifically validated rest intervals were strictly maintained, including 1-min passive recovery between the explosive power trials, 3-min active recovery between the test categories, and extended 10-min recovery periods following the completion of all physical quality evaluations.

The second phase involved specialized technique assessments following the identical warm-up protocol. The technical parameters evaluated included ankle joint cushioning amplitude, hip joint angle at the moment of landing on the net, time from the lowest point to the start of the push-off, counter-jump height/pre-jump height ratio, net depth/weight ratio, horizontal displacement, and angular velocity of arm swinging. Between the trial sets, a standardized 7-min cryotherapy interval was implemented. The final phase systematically evaluated perceptual ability and psychological quality through a series of tests, including 5-s time perception, 10-s time perception, three self-rotations, 10 self-rotations, standing straight-leg raise at 45 degrees, supine straight-leg raise at 45 degrees, and foot dorsiflexion to 20 cm, as well as assessments of state anxiety, trait anxiety, daily acrophobic sensations and responses, and trampoline-induced acrophobic sensations and responses.

All testing procedures incorporated rigorous quality control measures, including standardized administration protocols and scientifically determined recovery periods to ensure data reliability.

In the test of anthropometry indicators, a standardized measurement procedure was used to obtain data such as height, weight, BMI, limb length, and width. Height was measured to the nearest 0.1 cm using a wall-mounted stadiometer (GMCS-I, Jianmin, China), with the participants maintaining the Frankfort plane orientation, while weight was recorded using calibrated digital scales (BLK-600, Beilu, China; precision ±0.1 kg) for BMI calculation. Limb dimensions included leg length (measured from the anterior superior iliac spine to the medial malleolus) and upper limb length (from the acromion to the radial styloid process), both measured with anthropometric tape. Shoulder width (acromion–acromion distance) and hip width (maximum trochanteric breadth) were measured using sliding calipers. Circumferential measurements at the midpoints of the thigh (inguinal crease–patella midpoint), calf (maximum gastrocnemius protrusion), chest (nipple line), and waist (narrowest abdominal point during expiration) were taken with non-elastic tape. All measurements were performed by trained researchers in accordance with the ISAK standards, with duplicate trials conducted when variance exceeded 5%, and daily instrument calibration ensured data reliability.

For the assessment of physical quality, national fitness testing equipment (HK6800 series, Shenzhen Hengkang Jiaye Technology Co., Ltd., China) was used to assess the indicators of counter jump, squat, rebound after a high landing, sit-and-reach, and standing long jump. Stopwatches were used to assess the indicators of the 30-s hanging leg raise, 30-s burpees, 30-s back extension test, and 30-s sit-up and reach test. The arm hang angle was measured using protractors.

A JVC GC-PX10 high-speed camera, with a video shooting frequency of 125 frames per second, was used to record the pre-jump movements of the athletes. The videos of the movements were analyzed using video analysis software to obtain specialized technique indicators, including ankle joint cushioning amplitude, hip joint angle at the moment of landing on the net, time from the lowest point to the start of the push-off, the ratio of counter-jump height to pre-jump height, the ratio of net depth to weight, horizontal displacement, and angular velocity of arm swinging. Psychological indicators of state anxiety and trait anxiety were measured using the State-Trait Anxiety Inventory (STAI) questionnaire (Carlucci et al., 2023), while acrophobia scoring was measured using the Acrophobia Response Scale (Arroll et al., 2017).

### 2.5 Streamlining the preliminary set of indicators

Factor analysis was used to streamline the preliminary set of indicators. The steps of indicator streamlining were as follows: First, the KMO test and Bartlett's test of sphericity were used to verify the applicability of the data, ensuring that the KMO value was >0.5 and the Bartlett test significance was < 0.05. Second, principal component analysis was used to extract the main factors, and the maximum variance method was used for factor rotation to obtain a clearer factor structure. In the factor analysis, factors with a characteristic value >1 were selected as effective factors. Indicators with high load (generally >0.4) were extracted from the factor load matrix as representative indicators for each dimension to form the final indicator system of pre-jump height influencing factors in juvenile trampoline gymnasts.

### 2.6 Pearson correlation analysis and grey relational analysis

Pearson correlation analysis between pre-jump height and each indicator of pre-jump height influencing factors was performed using SPSS version 22.0 for Windows (SPSS, Inc. Chicago, IL, United States). For the grey relational analysis, the indicators of pre-jump height influencing factors served as inspection sequences and pre-jump height was designated as the standard sequence. The normalization of each standard and inspection sequence data was performed by dividing the average value of the sequence. We then calculated the grey relational coefficient using the pre-processed sequences and Deng's formula for grey relational grade. The formula is shown in Equation 1.


(1) $$\begin{array}{l} corr\left(\text{x}0\left(\text{k}\right),\text{ xi}\left(\text{k}\right)\right)=\frac{\text{Δ}\min+p\text{Δ}\max}{\text{Δ}0\text{i}\left(\text{k}\right)+\text{pΔ}\max} \end{array}$$

Where i = 1, 2, 3, …, m and k = 1, 2, 3, …, n; x0 and xi indicate the standard sequence and inspected sequence, respectively. Δ0i = ||x0(k) – xi(k)|| is the difference between x0 and xi. Δmin = ∀imin.min. ∀k||x0(k) – xi(k)|| and Δmax = ∀imax.max. ∀k||x0(k) – xi(k)||. *p* is the distinguishing coefficient and *p*ε[0,1]. According to previous research, we used *p* = 0.5 in this study.

To calculate the grey correlation grade (CORR), the mean value of the grey correlation coefficients was computed, as described by the following equation:


(2) $$\begin{array}{l} \text{CORR}\left({\text{x}}_{0},{\text{x}}_{\text{i}}\right)=\frac{1}{n}\;\overset{n}{\underset{k=1}{\sum }}corr\left(\text{x}0\left(\text{k}\right),\text{xi}\left(\text{k}\right)\right) \end{array}$$

In this study, the grey correlation grade (CORR) ranged from 0 to 1. A higher CORR value indicates a closer trend of change between the EMG index and power or cadence, reflecting a stronger influence on exercise performance.

MATLAB R2016 software (MathWorks, Natick, MA, USA) was used for data processing.

## 3 Results

### 3.1 The set of predictive indicators for pre-jump height influencing factors

The set of predictive indicators for factors influencing pre-jump height in juvenile trampoline gymnasts constructed in this study included five primary indexes and 43 secondary indexes, including 13 anthropometry indicators, 10 physical quality indicators, seven specialized technique indicators, seven perceptual ability indicators, and six psychological quality indicators, as shown in Table 1.

**Table 1:** The set of predictive indicators for factors influencing pre-jump height in juvenile trampoline gymnasts.

**Primary index**	**Secondary index**
Anthropometry	Height, weight, BMI, leg length, upper limb length, foot length, thigh circumference, calf circumference, chest circumference, waist circumference, shoulder width, hip width, and arm span.
Physical quality	Counter jump, squat, rebound after a high landing, sit-and-reach, standing long jump, 30-s hanging leg raise, 30-s burpees, 30-s back extension test, 30-s sit-up and reach test, and arm hang angle.
Specialized techniques	Ankle joint cushioning amplitude, hip joint angle at the moment of landing on the net, time from the lowest point to the start of the push-off, counter-jump height/pre-jump height ratio, net depth/weight ratio, horizontal displacement, and angular velocity of arm swinging
Perceptual ability	5-s time perception, 10-s time perception, three self-rotations perception, 10 self-rotations perception, standing straight-leg raise at 45 degrees perception, supine straight-leg raise at 45 degrees perception, and foot dorsiflexion to 20 cm perception.
Psychological quality	State anxiety, trait anxiety, daily acrophobic sensations, daily acrophobic responses, trampoline-induced acrophobic sensations, and trampoline-induced acrophobic responses

Factor analysis was conducted on the preliminary indicators of anthropometry, which resulted in four principal components: body dimension factor, body stability factor, height and weight factor, and lower limb length scale factor. The body dimension factor was represented by BMI, the body stability factor by shoulder width, the height and weight factor by height, and the limb length scale factor by the ratio of leg length to height. The results are shown in Table 2.

**Table 2:** The refined results of the anthropometry indicators for juvenile trampoline gymnasts.

**Code**	**Contribution**	**Principal component**	**Representative indicators**	**Other indicators**
1	34.259	Body dimension factor	BMI	Arm span to height ratio, thigh circumference, calf circumference, chest circumference, and waist circumference
2	16.633	Body stability factor	Shoulder width	Hip width, foot length
3	12.333	Height and weight factor	Height	Weight
4	10.608	Limb length scale factor	Leg length	Upper limb length
KMO = 0.748		Cumulative = 73.83%	Bartlett's Sphericity Test P < 0.001	

Table 3 presents the refined results of the physical quality indicators for juvenile trampoline gymnasts. As shown in Table 3, factor analysis of the preliminary physical quality indicators extracted three principal components: Lower limb and back muscle strength factor, flexibility and abdominal muscle strength factor, and arm hang angle factor. The lower limb and back muscle strength factor was represented by the indicator standing long jump. The flexibility and abdominal muscle strength factor was represented by the indicator 30-s hanging leg raise. The arm hang angle factor was represented by the indicator arm hang angle.

**Table 3:** The refined results of the physical quality indicators for juvenile trampoline gymnasts.

**Code**	**Contribution**	**Principal component**	**Representative indicators**	**Other indicators**
1	42.192	Lower limb and back muscle strength factor	Standing long jump	30-s back extension test, 30-s burpees, counter jump, squat, 30-s sit-up and reach
2	18.741	Flexibility and abdominal muscle strength factor	30-s hanging leg raise	Rebound after a high landing, sit-and-reach
3	11.873	Arm hang angle factor	Arm hang angle	
KMO = 0.690		Cumulative = 72.81%	Bartlett's Sphericity Test P < 0.001	

The refined results of the specialized technique indicators for juvenile trampoline gymnasts are shown in Table 4. A total of three principal components of the specialized technique indicators were extracted: Net pressing strength factor, shoulder and ankle joint control factor, and trampoline control factor. The net pressing strength factor was represented by the hip joint angle at the moment of landing on the net, the shoulder and ankle joint control factor by the ankle joint cushioning amplitude, and the trampoline control factor by the ratio of counter-jump height to pre-jump height.

**Table 4:** The refined results of specialized technique indicators for juvenile trampoline gymnasts.

**Code**	**Contribution**	**Principal component**	**Representative indicators**	**Other indicators**
1	29.72	Net pressing strength factor	Hip joint angle at the moment of landing on the net	Net depth/weight ratio
2	23.24	Shoulder and ankle joint control factor	Ankle joint cushioning amplitude	Angular velocity of arm swinging
3	18.03	Trampoline control factor	Counter-jump height/pre-jump height ratio	Time from the lowest point to the start of the push-off
				Horizontal displacement
KMO = 0.686		Cumulative = 70.98%	Bartlett's Sphericity Test P < 0.001	

The refined results of the perceptual ability indicators for juvenile trampoline gymnasts are shown in Table 5. A total of three principal components of the perceptual ability indicators were extracted: Joint position sense factor, self-rotation angle perception factor, and time and distance perception factor. The joint position sense factor was represented by the supine straight-leg raise at 45 degrees perception indicator. The self-rotation angle perception factor was represented by the 10 self-rotations perception indicator. The time and distance perception factor was represented by the 10-s time perception indicator.

**Table 5:** The refined results of the perceptual ability indicators for juvenile trampoline gymnasts.

**Code**	**Contribution**	**Principal component naming**	**Representative indicators**	**Other indicators**
1	31.543	Joint position sense factor	Supine straight-leg raise at 45 degrees perception	Standing straight-leg raise at 45 degrees perception
2	21.304	Self-rotation angle perception factor	10 self-rotations perception	Three self-rotations perception
3	17.965	Time and distance perception factor	10-s time perception	5-s time perception, foot dorsiflexion to 20 cm perception
KMO = 0.558		Cumulative = 70.81%	Bartlett's Sphericity Test P = 0.021	

The refined results of the psychological quality indicators for juvenile trampoline gymnasts are shown in Table 6. Three principal components of the psychological quality indicators were extracted: Daily acrophobic factor, state-trait anxiety factor, and trampoline-induced acrophobic factor. The daily acrophobic factor was represented by the indicator of daily acrophobic sensations, the state trait anxiety factor by the indicator of state anxiety, and the trampoline-induced acrophobic factor by the indicator of trampoline-induced acrophobic sensations.

**Table 6:** Refined results of psychological quality indicators for juvenile trampoline gymnasts.

**Code**	**Contribution rate**	**Principal component**	**Representative indicators**	**Other indicators**
1	42.62	Daily acrophobic factor	Daily acrophobic sensations	Daily acrophobic responses
2	25.27	State-trait anxiety factor	State anxiety	Trait anxiety
3	16.75	Trampoline-induced acrophobic factor	Trampoline-induced acrophobic sensations	Trampoline-induced acrophobic responses
KMO = 0.514		Cumulative = 84.65%	Bartlett's Sphericity Test P < 0.001	

After factor analysis, a set of predictive indicators for factors influencing pre-jump height was obtained, as shown in Table 7. The test results for each representative indicator are shown in Table 8.

**Table 7:** Overview of the indicator system of pre-jump height influencing factors in juvenile trampoline gymnasts.

**Indicator category**	**Indicator name**
Body dimension factor	BMI
Body stability factor	Shoulder width
Height and weight factor	Height
Lower limb length scale factor	Leg length
Lower limb and back muscle strength factor	Standing long jump
Flexibility and abdominal muscle strength factor	30-s hanging leg raise
Arm hang angle factor	Arm hang angle
Net pressing strength factor	Hip joint angle at the moment of landing on the net
Shoulder and ankle joint control factor	Ankle joint cushioning amplitude
Trampoline control factor	Counter-jump height/pre-jump height ratio
Joint position sense factor	Supine straight-leg raise at 45 degrees perception
Self-rotation angle perception factor	10 self-rotations perception
Time and distance perception factor	10-s time perception
Daily acrophobic factor	Daily acrophobic sensations
State-trait anxiety factor	State anxiety
Trampoline-induced acrophobic factor	Trampoline-induced acrophobic sensations

**Table 8:** Test results for pre-jump height and each representative indicator.

**Ranking**	**Indicator name**	**Test results (mean ±SD)**
1	Leg length (ratio to height)	0.565 ± 0.011
2	BMI	17.23 ± 1.62
3	Height (cm)	152.66 ± 9.98
4	Daily acrophobic sensations (rating)	40.81 ± 18.60
5	Ankle joint cushioning amplitude (degree)	10.15 ± 5.09
6	Standing long jump (meter)	212.75 ± 17.64
7	Shoulder width (ratio to height)	0.195 ± 0.007
8	Arm hang angle (degree)	173.44 ± 5.15
9	Hip joint angle at the moment of landing on the net (degree)	21.14 ± 2.19
10	Counter-jump height/pre-jump height (ratio)	0.144 ± 0.015
11	30-s hanging leg raise (times)	19.69 ± 1.16
12	10-s time perception (seconds)	0.209 ± 0.144s
13	10 self-rotations perception (degree)	66.69 ± 78.17°
14	Supine straight-leg raise at 45 degrees perception (degree)	4.56 ± 4.15
15	State anxiety (score)	37.44 ± 6.13
16	Trampoline-induced acrophobic sensations (score)	13.31 ± 3.49
	Pre-jump height (m)	3.13 ± 0.36

### 3.2 Grey relational analysis of pre-jump height and each indicator of the pre-jump height influencing factors

Table 9 shows the results of the Pearson correlation analysis and grey relational coefficients between pre-jump height and each indicator of the pre-jump height influencing factors. A total of 10 indicators (e.g., standing long jump, height, leg length, arm hang angle) showed high grey relational coefficients (>0.9) with pre-jump height. The three indicators—trampoline-induced acrophobic sensations, daily acrophobic sensations, and ankle joint cushioning amplitude—had grey relational coefficients between 0.8 and 0.9 with pre-jump height. The indicators-−10-s time perception, supine straight-leg raise at 45 degrees perception, and 10 self-rotations perception—had a grey relational coefficient value of < 0.8 with pre-jump height. The changing trends observed in the correlation analysis results were generally consistent with those in the grey relational analysis.

**Table 9:** The results of the Pearson correlation analysis and grey relational coefficients between pre-jump height and each indicator of the pre-jump height influencing factors.

**Sort**	**Indicator name**	**Pearson correlation**	**Grey correlation degree**
1	Standing long jump	0.871^*^	0.9469
2	Height	0.840^*^	0.9428
3	Leg length	0.875^*^	0.9408
4	Shoulder width	0.762^*^	0.9369
5	Arm hang angle	0.627^*^	0.9345
6	Counter-jump height/pre-jump height ratio	0.754^*^	0.9316
7	30-s hanging leg raise	0.686^*^	0.9315
8	BMI	0.771^*^	0.9288
9	Hip joint angle at the moment of landing on the net	0.675^*^	0.9203
10	State anxiety	−0.631^*^	0.9116
11	Trampoline-induced acrophobic sensations	−0.554^*^	0.8659
12	Daily acrophobic sensations	0.495^*^	0.8274
13	Ankle joint cushioning amplitude	0.576^*^	0.8023
14	10-s time perception	−0.287	0.7486
15	Supine straight-leg raise at 45 degrees perception	−0.254	0.6732
16	10 self-rotations perception	−0.292	0.6574

^*^Represent the correlation between the indicator and pre-jump height is statistically significant at the 0.05 level (*p* < 0.05).

## 4 Discussion

The main results of the current study included two aspects: First, an indicator system of pre-jump height influencing factors was established in this study, aligning with the need for a comprehensive understanding of the factors affecting trampoline performance. Second, the impact of each indicator on pre-jump height was quantified and compared using the grey relational analysis method, and the key factors influencing pre-jump height in juvenile trampoline gymnasts were explored. To the best of our knowledge, this is one of the pioneering studies to explore the influencing variables and the degree of impact of each variable on pre-jump height in juvenile trampoline gymnasts.

### 4.1 Anthropometric profile

Previous research has extensively demonstrated significant associations between anthropometric characteristics and sport performance (Brocherie et al., 2014; Kaur and Koley, 2019). For example, in the study by Douda et al. (2008), anthropometric components were found to account for 45% of the total variance in rhythmic gymnastics performance and significantly correlate with sport performance (*r* = 0.5, *p* < 0.05) (Douda et al., 2008).

In the current study, the four anthropometric indicators—height, leg length, shoulder width, and BMI—had a grey relational coefficient higher than 0.9 with pre-jump height. The results may indicate close relationships between the four anthropometric indicators and pre-jump height in juvenile trampoline gymnasts. This is consistent with previous research highlighting the importance of anthropometric measures in sport performance (Fang, 2016; Feng and Li, 2009), suggesting that the anthropometry of juvenile trampoline gymnasts may have a significant impact on pre-jump height (de Oliveira et al., 2021; Zhang and He, 2007; Jiao, 2006).

### 4.2 Physical quality profile

In this study, three indicators of physical quality were adopted. The three physical quality indicators—standing long jump, 30-s hanging leg raise, and arm hang angle—had a grey relational coefficient higher than 0.9 with pre-jump height. The results may indicate close relationships between the three indicators and pre-jump height in juvenile trampoline gymnasts. This is consistent with previous research highlighting the importance of physical qualities in trampoline performance (Uçan, 2018; Sun et al., 2024). The standing long jump indicator reflects the lower limb explosive power and body coordination required, making it a key physical quality for achieving an excellent pre-jump height (Lindberg et al., 2024). The 30-s hanging leg raise indicator reflects the athlete's waist and abdominal muscle endurance, coordination, and body flexibility, all of which are important physical quality indicators for trampoline athletes (Dyas et al., 2023; Potop et al., 2014; McGill et al., 2015; Gao, 2023). As a unique physical quality indicator for trampoline athletes, the arm raise angle plays a vital role in adjusting body posture and the center of gravity during trampoline performance and is an important factor affecting jump height (Hara et al., 2005). The arm raise angle is an important factor affecting the position of the athlete's center of gravity and the direction of the trampoline's rebound force when landing on the net. Based on biomechanical theory analysis, when the arm raise angle is 180 degrees, it ensures that the angle between the rebound force of the net surface and the vertical direction is smaller, thereby making it more conducive to achieving optimal landing performance on the net (Winter, 2009).

### 4.3 Specialized technique profile

Trampoline gymnastics is a highly technical sport, and the quality of techniques performed on the trampoline bed directly determines athletic performance. Among these techniques, the ability of athletes to effectively utilize the elasticity of the trampoline bed during preliminary jumps is a crucial skill (Qian et al., 2020). In this study, three indicators of specialized techniques including the ratio of counter-jump height/pre-jump height, hip joint angle at the moment of landing on the net, and ankle joint cushioning amplitude were adopted. The grey relational coefficients between pre-jump height and the two indicators—the ratio of counter-jump height/pre-jump height and the hip joint angle at the moment of landing on the net—were all higher than 0.9, while the indicator of ankle joint cushioning amplitude during landing on the trampoline had a grey relational coefficient between 0.8 and 0.9 with pre-jump height. The results revealed the significant role of specialized techniques in influencing the performance of pre-jump height.

The ratio of counter-jump height/pre-jump height may demonstrate the ability to control and utilize the elasticity of the trampoline surface to achieve maximum pre-jump height and is essential for the competitive ability of trampoline athletes (Qian et al., 2020). The hip joint angle at the moment of landing on the net is related to the effect of rapid hip extension for pressing the net surface at the moment of landing, and to some extent, it reflects the net pressure force at the beginning of net touch (Diener-Gonzalez and Aedo-Munoz, 2017). It has been suggested that a greater ankle joint cushioning amplitude during landing on the trampoline may provide a larger range of motion for force production during the rapid extension phase. This helps generate a greater extension impulse and thus can lead to an increased take-off velocity and enhanced pre-jump height.

### 4.4 Perceptual ability and psychological quality profile

In this study, five indicators of perceptual ability and one indicator of psychological quality were adopted. The grey relational coefficient between the level of state anxiety and pre-jump height was higher than 0.9, indicating a close relationship between the level of state anxiety and pre-jump height. The level of state anxiety reflects the tension and anxiety experienced by athletes before training and competitions, which can have a significant impact on competitive performance and pre-jump height (Ong and Chua, 2021).

The two indicators—trampoline-induced acrophobic sensations and daily acrophobic sensations—had a grey relational coefficient between 0.8 and 0.9 with pre-jump height. The inclusion of trampoline-induced acrophobic sensations and daily acrophobic sensations may suggest that the fear of height during trampoline sports may be an important factor influencing pre-jump height and competitive performance. Therefore, the intervention of acrophobic sensation training is needed in practical sessions for juvenile trampoline gymnasts (Yang et al., 2012). In addition to traditional methods such as psychological counseling and relaxation training, using virtual reality (VR) technology for simulated training may be an effective approach to help athletes experience and adapt to the sensation of height, thereby addressing acrophobia in trampoline gymnasts (Suyanto et al., 2017; Bürger et al., 2022).

The 10-s time perception, supine straight-leg raise at 45 degrees perception, and 10 self-rotations perception indicators had a grey relational coefficient value of < 0.8 with pre-jump height, indicating that the perceptual ability of juvenile trampoline gymnasts represented by the three indicators may also have a certain impact on pre-jump height, although their influence is less significant than that of the aforementioned 13 indicators (Ferger et al., 2019; Gautier et al., 2007).

### 4.5 Limitations of the study

The following limitations of this study should be acknowledged: (1) Due to the restrictions imposed by practical conditions and professional training teams, the number of adolescent trampoline athletes selected for the study was relatively small, which might have affected the reliability, validity, and generalizability of the research findings. (2) The wide age range of the participants in the current study might have resulted in the research findings being influenced by age-related factors. (3) In the measurement of specialized technique indicators, the use of video analysis might have introduced errors and affected the accuracy of the measurement results. (4) Measuring indicators such as athletes' anxiety levels and trampoline-induced acrophobic sensations through questionnaire surveys might have been affected by differences in individual judgment standards.

### 4.6 Practical applications

Identifying the key influencing factors of pre-jump height in juvenile trampoline gymnasts has important implications for practitioners. First, this information helps practitioners develop targeted training programs and better track and evaluate both the results and the process of training for juvenile trampoline gymnasts. Second, the results of the current study may provide scientific recommendations for developing objective talent selection criteria and improving the selection process of trampoline athletes. Third, these findings may also advance gymnastics research by highlighting the interplay of physical, technical, and psychological factors in specialized jumping performance.

However, in this study, the grey relational coefficient between each influencing indicator and pre-jump height was calculated using data from all tested athletes collectively. It reflects the average impact of various influencing factors on the pre-jump height of juvenile trampoline gymnasts to a certain extent. For a specific athlete, the importance of each indicator on pre-jump height may differ; therefore, individualized diagnosis and evaluation of each indicator may be needed in practical training.

## 5 Conclusion

This study established an indicator system consisting of 16 items of pre-jump height influencing factors and identified the importance ranking of each indicator using grey relational analysis in juvenile trampoline gymnasts. These findings may serve as a scientific basis for developing targeted training programs and objective talent selection criteria while also advancing gymnastics research by highlighting the interplay of physical, technical, and psychological factors in specialized jumping performance.

## Data Availability

The original contributions presented in the study are included in the article/supplementary material, further inquiries can be directed to the corresponding authors.
